# Experimental Cannabinoid 2 Receptor-Mediated Immune Modulation in Sepsis

**DOI:** 10.1155/2014/978678

**Published:** 2014-04-03

**Authors:** J. Sardinha, M. E. M. Kelly, J. Zhou, C. Lehmann

**Affiliations:** ^1^Department of Pharmacology, Dalhousie University, Halifax, NS, Canada B3H 4R2; ^2^Department of Anesthesia, Dalhousie University, Halifax, NS, Canada B3H 2Y9; ^3^Department of Microbiology & Immunology, Dalhousie University, Halifax, NS, Canada B3H 4R2

## Abstract

Sepsis is a complex condition that results from a dysregulated immune system in response to a systemic infection. Current treatments lack effectiveness in reducing the incidence and mortality associated with this disease. The endocannabinoid system offers great promise in managing sepsis pathogenesis due to its unique characteristics. The present study explored the effect of modulating the CB_2_ receptor pathway in an acute sepsis mouse model. Endotoxemia was induced by intravenous injection of lipopolysaccharide (LPS) in mice and intestinal microcirculation was assessed through intravital microscopy. We found that HU308 (CB_2_ receptor agonist) reduced the number of adherent leukocytes in submucosal venules but did not restore muscular and mucosal villi FCD in endotoxemic mice. AM630 (CB_2_ receptor antagonist) maintained the level of adherent leukocytes induced by LPS but further reduced muscular and mucosal villi FCD. URB597 (FAAH inhibitor) and JZL184 (MAGL inhibitor) both reduced the number of adherent leukocytes in submucosal venules but did not restore the mucosal villi FCD. Using various compounds we have shown different mechanisms of activating CB_2_ receptors to reduce leukocyte endothelial interactions in order to prevent further inflammatory damage during sepsis.

## 1. Introduction

Sepsis and septic shock are the leading causes of mortality in intensive care units worldwide [1]. Globally an estimated 19 million cases of sepsis occur per year, with one-third of the patients dying from the condition [2, 3]. Sepsis is a complex immune syndrome characterized by an imbalance between pro- and anti-inflammatory mediators systemically released in high amounts (cytokine storm) in response to an infection [4, 5]. During the early stages of sepsis, immune cells are often hyperactivated and may lose their ability to differentiate between infectious targets and healthy cells (horror autotoxicus). If left untreated, consequences of immune system dysregulation include impairment of circulatory function (septic shock), leading to subsequent poor tissue perfusion. Eventually, organ systems start failing from lack of nutrients leading to patient mortality [6]. Current treatments for sepsis include administration of antibiotics to fight the infection and fluid resuscitation and vasopressors to combat hypotension. However, there are no approved treatment options available that target the malfunctioning immune system [7].

The endocannabinoid system has recently emerged as a potential target in sepsis treatment [8]. This system is an endogenous signalling system that mediates a variety of physiological functions including modulation of the immune system. The endocannabinoid system is composed of endogenous ligands (endocannabinoids), cannabinoid receptors, and enzymes that synthesize and degrade endocannabinoids [9]. The two most well-known endocannabinoids are arachidonoylethanolamide (AEA; formerly known as anandamide) and 2-arachidonoylglycerol (2-AG) [10, 11]. These ligands activate a variety of receptors, but the two most important are the G-protein coupled cannabinoid 1 (CB_1_) and cannabinoid 2 (CB_2_) receptors [12]. CB_1_ receptors are found throughout the body, including in the central nervous system. These receptors mediate the psychotropic actions of the Δ^9^-THC, a phytocannabinoid constituent of* Cannabis sativa*. CB_2_ receptors are strongly expressed on the surface of immune cells [13, 14]. It has been well documented that activation of the CB_2_ receptors causes an immunosuppressive response [15, 16]. The effects of AEA and 2-AG are locally ablated by degradation through two enzymes, fatty acid amide hydrolase (FAAH) [17] and monoacylglycerol lipase (MAGL), respectively [18].

The aim of our study was to elucidate the potential role of CB_2_-mediated immune modulation in sepsis with a focus on the intestinal microcirculation that plays a critical role in the pathophysiology of sepsis [19, 20]. During the initial stages of sepsis, a significant proinflammatory response occurs evidenced by marked increases in leukocyte endothelial interactions. We therefore investigated changes in leukocyte recruitment as well as functional capillary density in mice challenged with endotoxin as an acute experimental model of sepsis (endotoxemia). Previous studies have suggested a beneficial role of CB_2_ receptor activation in attenuating leukocyte endothelial interactions, as well as proinflammatory mediators [21–24]. These studies have shown that CB_2_ receptor activation can be beneficial in inflammatory states by reducing release of proinflammatory cytokines like TNF-*α*, activation of endothelial cells, transmigration of inflammatory infiltrates, and reactive damage by oxidative stress and apoptosis. Our experiments examined the effects of CB_2_ receptor modulation on intestinal microcirculation by using CB_2_ agonists and antagonists and inhibitors of FAAH and MAGL.

## 2. Methods

### 2.1. Animals

Male C57BL/6 mice (6 weeks old; 20–30 g) were purchased from Charles River Laboratories International Inc. (Wilmington, MS, USA). Animals were housed in ventilated rack cages and allowed to acclimatize for a week at the Carleton animal care facility of the Faculty of Medicine at Dalhousie University, Halifax, NS, Canada. Animals were kept on a standard 12-hour light/dark cycle, with standard room temperature 22°C and humidity 55%–60%. Animals were fed a standard diet of rodent chow and water* ad libitum*. This study was conducted in accordance with the guidelines and standards set forth by the Canadian Council on Animal Care and approved by the University Committee on Laboratory Animals at Dalhousie University.

### 2.2. Endotoxemia

Animals were anesthetised with 90 mg/kg pentobarbital (Ceva Sante Animale, Montreal, QC, Canada) administered intraperitoneally (I.P.) and supplemented with 20 mg/kg pentobarbital intravenously (I.V.) during the experiment when needed. Animals breathed room air spontaneously, but oxygen was provided if breathing got laboured. The mouse was placed on a heating pad in supine position to maintain body temperature at 37°C (98.6°F) and a rectal temperature probe was used for the measurement. The left jugular vein was cannulated with polyethylene tubing (PE 10, Clay Adams, Sparks, MD, USA) for administration of fluids and drugs.

### 2.3. Experimental Groups

Six groups of animals were examined (*N* = 3–5 per group). Group 1 served as control (CON) group which only received saline (0.9% sodium chloride, Hospira, Montreal, QC, Canada) at an equal volume of LPS as described below. All other groups received an I.V. dose of 5 mg/kg lipopolysaccharide (LPS,* Escherichia coli*, serotype O26:B6, Sigma-Aldrich, Oakville, ON, Canada). Group 2 received no further treatment besides LPS. Groups 3–6 received treatment compounds administered I.V. 15 minutes after administration of LPS. Group 3 received a CB_2_ receptor agonist, HU308 (2.5 mg/kg; Tocris Bioscience, Ellisville, MO, USA). Group 4 received a CB_2_ receptor antagonist/inverse agonist, AM630 (2.5 mg/kg; Tocris Bioscience, Ellisville, MO, USA). Group 5 received a FAAH inhibitor, URB597 (0.6 mg/kg; Tocris Bioscience, Ellisville, MO, USA). Group 6 received a MAGL inhibitor, JZL184 (16 mg/kg; Tocris Bioscience, Ellisville, MO, USA). All treatment compounds were dissolved in dimethyl sulfoxide (DMSO) and stock solutions were further diluted with saline to a final concentration of 10% (30% for AM630). Pilot experiments did not show detrimental effects within the microcirculation using DMSO concentrations up to 50%. 

### 2.4. Intravital Microscopy

Intravital microscopy (IVM) of the terminal ileum was performed using an epifluorescent microscope (Leica DMLM, Wetzlar, Germany) with a mercury-arc light source (LEG EBQ 100; Carl Zeiss, Jena, Germany). Videos were recorded on a standard personal computer using IC capture software (v2.2, Imaging Source; Charlotte, NC, USA) and stored on external hard drives. To access the intestine, an abdominal midline incision was made. Using saline soaked cotton tipped applicators, a section of the terminal ileum was exposed and placed on a specially designed apparatus [25]. Using this apparatus, a liquid contact is made between the intestine and the cover slip. This technique minimizes pressure on the tissue in addition to the added benefit of constantly hydrating the intestine with thermostat controlled saline (37°C/98.6°F).

To observe leukocyte activation within the intestinal microvasculature, animals were intravenously administered 0.05% rhodamine 6G-solution (1.5 mL/kg, Sigma-Aldrich). Leukocyte adherence was observed in collecting venules (V1; >50 *μ*m diameter) as well as postcapillary venules (V3; <50 *μ*m diameter). Leukocytes that remained immobile on the endothelium for 30 seconds qualified as adherent leukocytes, while all nonadherent leukocytes patrolling past a designated point across the vessel were quantified as rolling leukocytes. This data allowed us to estimate the number of adherent leukocytes (cells/mm^2^), as well as the number of rolling leukocytes (cells/minute), along the intestinal endothelium. To observe microvascular integrity, animals were intravenously administered 5% fluorescein isothiocyanate- (FITC-) tagged albumin (1 mL/kg; Sigma-Aldrich). Capillary perfusion in the muscle layers and the mucosal villi of the intestine was observed. To visualize the mucosal villi, the intestinal surface was cauterized and cut to expose the lumen. The length of perfused capillaries was measured in a defined area and used to calculate the functional capillary density (FCD, *μ*m/*μ*m^2^). Six visual fields of each vascular type were recorded for 30 seconds each.

### 2.5. Statistical Analysis

Results were analyzed by using the software Prism 5 (GraphPad Software, La Jolla, CA, USA). All data were analysed using a one-way ANOVA with a post hoc Newman-Keuls correction. All data are expressed as means ± standard deviation (SD). Significance was set at *P* < 0.05.

## 3. Results

### 3.1. Leukocyte Adhesion

**Figure 1:**
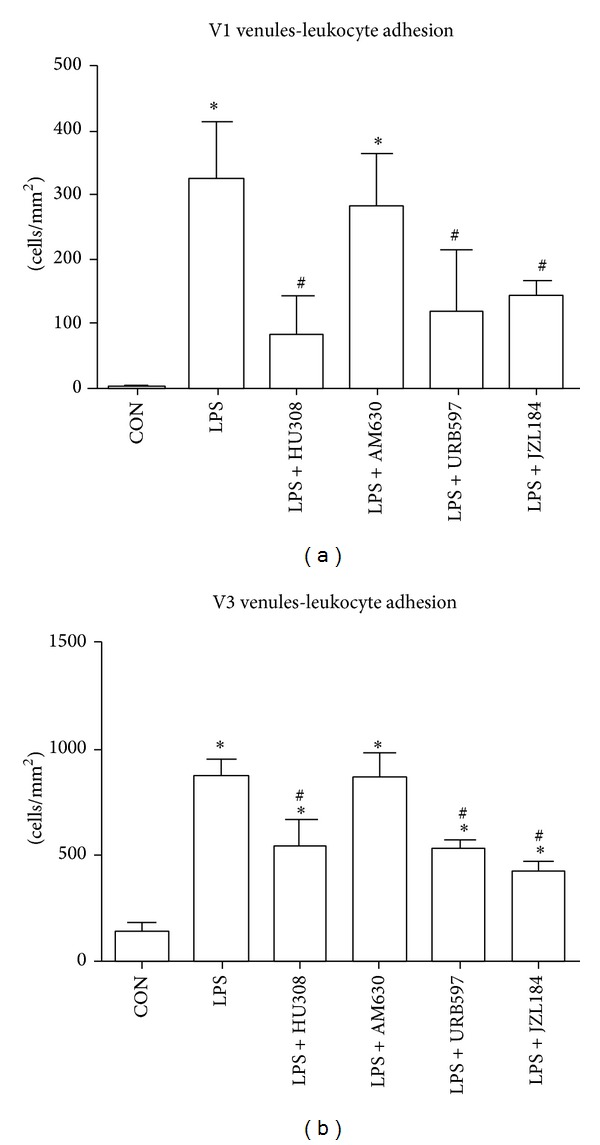
(a) Adherent leukocytes in collecting venules (V1; >50 *μ*m vessel diameter). (b) Adherent leukocytes in postcapillary venules (V3; <50 *μ*m vessel diameter). Control group (CON); endotoxemia group LPS (5 mg/kg); LPS + HU308 (2.5 mg/kg), a CB_2_ receptor agonist; LPS + AM630 (2.5 mg/kg), a CB_2_ receptor antagonist/inverse agonist; LPS + URB597 (0.6 mg/kg), a FAAH inhibitor; and LPS + JZL184 (16 mg/kg) a MAGL inhibitor. In every animal six V1 venules and six V3 venules were analyzed (*n* = 3–5 mice/group). Data presented as mean ± standard deviation. *P < 0.05 versus control. ^#^ *P* < 0.05 versus LPS.

Endotoxin challenge significantly (*P* < 0.05) increased the number of adherent leukocytes in V1 and V3 venules compared to controls (Figures 1(a) and 1(b)). They showed a 100-fold increase in V1 venules and a 10-fold increase in V3 venules.

Administration of HU308, URB597, or JZL184 after LPS challenge significantly (*P* < 0.05) reduced the number of adherent leukocytes in V1 and V3 venules in comparison to untreated LPS animals (Figures 1(a) and 1(b). These treatments following LPS challenge reduced the number of adherent leukocytes to the same levels as the non-LPS-challenged control group in V1 (Figure 1(a); *P* > 0.05) but not in V3 venules (Figure 1(b); *P* < 0.05). Administration of AM630 after LPS challenge showed no significant (*P* > 0.05) difference in leukocyte adherence to LPS group in V1 and V3 venules (Figures 1(a) and 1(b)).

### 3.2. Leukocyte Rolling

**Figure 2:**
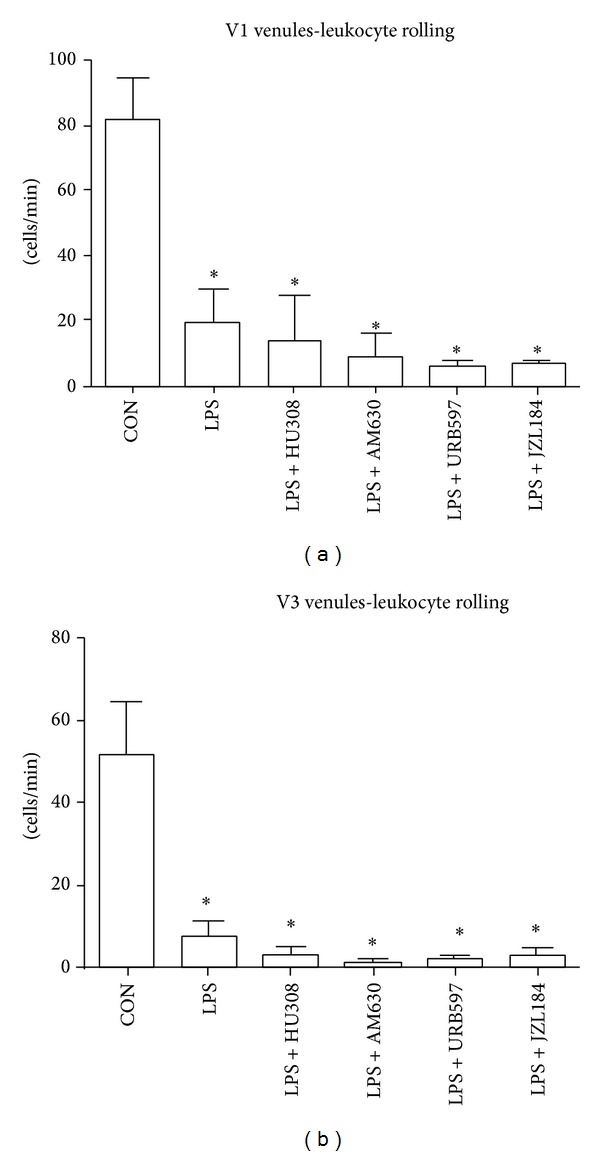
(a) Rolling leukocytes along collecting venules (V1; >50 *μ*m vessel diameter). (b) Rolling leukocytes along postcapillary venules (V3; <50 *μ*m vessel diameter). Control group (CON); endotoxemia group LPS (5 mg/kg); LPS + HU308 (2.5 mg/kg), a CB_2_ receptor agonist; LPS + AM630 (2.5 mg/kg), a CB_2_ receptor antagonist/inverse agonist; LPS + URB597 (0.6 mg/kg), a FAAH inhibitor; and LPS + JZL184 (16 mg/kg), a MAGL inhibitor. In every animal six V1 venules and six V3 venules were analyzed (*n* = 3–5 mice/group). Data presented as mean ± standard deviation. *P < 0.05 versus control.

In comparison to controls, LPS-challenged animals showed a significant (*P* < 0.05) reduction in the number of rolling leukocytes for both V1 and V3 venules (Figures 2(a) and 2(b)). Administration of either HU308, AM630, URB597, or JZL184 after LPS challenge did not change the number of rolling leukocytes in comparison to LPS alone group in V1 venules (Figure 2(a)) and V3 venules (Figure 2(b)).

### 3.3. Functional Capillary Density (FCD)

**Figure 3:**
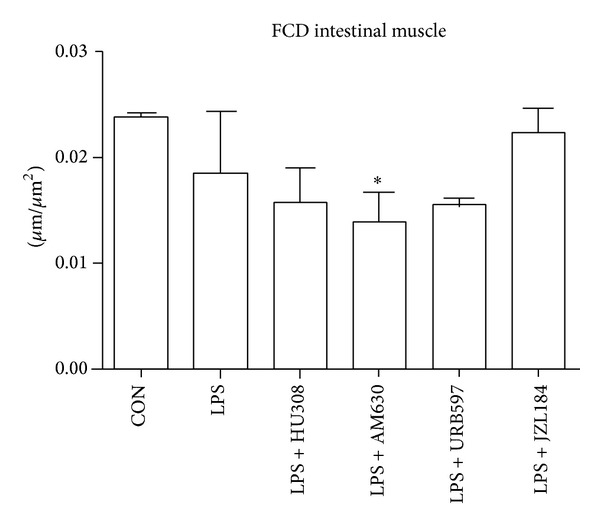
Functional capillary density (FCD) as a measure of capillary perfusion within the muscle layers of the intestine. Calculated as total length of capillaries with erythrocyte perfusion within a predetermined rectangular field. Control group (CON); endotoxemia group LPS (5 mg/kg); LPS + HU308 (2.5 mg/kg), a CB_2_ receptor agonist; LPS + AM630 (2.5 mg/kg), a CB_2_ receptor antagonist/inverse agonist; LPS + URB597 (0.6 mg/kg), a FAAH inhibitor; and LPS + JZL184 (16 mg/kg), a MAGL inhibitor. In every animal six intestinal muscle regions were analyzed (*n* = 3–5 mice/group). Data presented as mean ± standard deviation. *P < 0.05 versus control.

**Figure 4:**
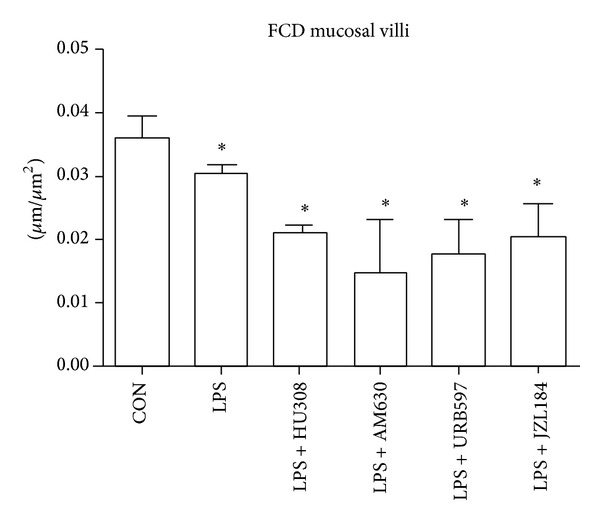
Functional capillary density (FCD) as a measure of capillary perfusion within mucosal villi. Calculated as total length of capillaries with erythrocyte perfusion within a predetermined rectangular field. Control group (CON); endotoxemia group LPS (5 mg/kg); LPS + HU308 (2.5 mg/kg), a CB_2_ receptor agonist; LPS + AM630 (2.5 mg/kg), a CB_2_ receptor antagonist/inverse agonist; LPS + URB597 (0.6 mg/kg), a FAAH inhibitor; LPS + JZL184 (16 mg/kg), a MAGL inhibitor. In every animal six mucosal regions were analyzed (*n* = 3–5 mice/group). Data presented as mean ± standard deviation. *P < 0.05 versus control.

Muscular functional capillary density showed no significant differences between controls and LPS (Figure 3). LPS + AM630 showed a significant reduction in muscle layer FCD compared to control. All other treatment groups showed no significant differences in muscular FCD when compared to controls or LPS. Mucosal FCD showed a significant reduction for all groups when compared to controls (Figure 4).

## 4. Discussion

In our study we compared for the first time the impact of various approaches to activate the CB_2_ receptor pathway in regard to leukocyte activation and functional capillary density within the intestinal microcirculation, which is a critical component in sepsis pathophysiology. We demonstrated the benefit of the CB_2_ receptor agonist, HU308, in reducing LPS-induced leukocyte recruitment. Furthermore, an alternate mechanism of activating the CB_2_ pathway through the enzyme inhibitors URB597 (FAAH) and JZL184 (MAGL) produced similar results. In contrast, blocking CB_2_ receptors by CB_2_ receptor antagonist AM630 caused FCD reduction in intestinal musculature.

Our endotoxemic model produced a robust immune response by increasing the number of adherent leukocytes within V1 and V3 venules and reducing the number of rolling leukocytes. LPS also affected capillary function as the FCD for the mucosal villi was reduced, but muscular FCD was not significantly lowered indicating an initiation of vascular damage. Our first therapeutic approach examined the effects of a CB_2_ receptor agonist HU308 on the intestinal microcirculation. Studies using CB_2_ agonists have indicated protective effects against inflammatory damage in various organs [14, 23]. They have shown reduction in expression of adhesion molecules such as intercellular adhesion molecule (ICAM) and vascular cell adhesion molecule (VCAM) [21, 24], reduction in levels of proinflammatory cytokines such as tumor necrosis factor-*α* (TNF-*α*) [21, 26], and decreased neutrophil infiltration but increased neutrophil activation [27]. Our results showed that HU308 was able to reduce the number of LPS-induced adherent leukocytes in V1 and V3 venules; however, the number of rolling leukocytes did not return to the levels seen in controls. These results suggest that HU308 suppresses leukocyte recruitment alluding to the lack of rolling leukocytes observed. HU308 treatment showed no improvement on mucosal villi FCD, indicating that this treatment did not have a sufficient impact on the proinflammatory mediators in preventing vascular damage. In general, HU308 administration showed some benefit after LPS administration by reducing the number of adherent leukocytes in both V1 and V3 venules, adding some support to other studies using CB_2_ agonist administration which show a reduction in leukocyte chemotaxis, endothelial interaction and transmigration, and release of proinflammatory mediators in experimental models of endotoxemia [26–29].

In order to further verify our findings that CB_2_ receptor activation through HU308 reduces LPS-induced microcirculatory damage, we blocked CB_2_ receptor activation with a CB_2_ receptor antagonist/inverse agonist AM630. Other studies have shown exacerbated inflammatory damage in their disease model with the use of AM630 [30] and neutralized therapeutic effects of AM630 in combination with a CB_2_ ligand [31, 32]. Our results correspond with the expected outcomes as AM630 maintained the number of adherent leukocytes induced by LPS. It is possible that maximal leukocyte recruitment may have been achieved after LPS challenge and therefore administration of AM630 following LPS could not further elevate leukocyte adherence. Furthermore, mucosal villi FCD was reduced and AM630 was the only group showing reduction in the muscular layers. It is possible that inflammatory processes were elevated beyond LPS-induced levels, indicated by a reduction in muscular FCD, even though exacerbated inflammation was not evident through leukocyte endothelial interactions.

Another therapeutic approach to activate the CB_2_ pathway is through inhibition of endocannabinoid hydrolysing enzymes. Inhibiting enzymes that degrade endocannabinoids can cause elevated levels of the endogenous compounds AEA and 2-AG, resulting in prolonged stimulation of the CB_2_ receptors. Using various synthetic compounds and FAAH deficient mice, researchers have shown that FAAH inhibition reduced hydrolysis rates for AEA, reduced pain sensitivity and analgesia, and reduced inflammation, all indicative of elevated AEA levels and cannabinoid receptor activation [33–36]. Our results with an irreversible FAAH inhibitor URB597 indicated analogous findings to HU308 administration: leukocyte endothelial interactions were reduced in both submucosal V1 and V3 venules, and FCD was not improved in muscular and mucosal villi. Previous studies in our laboratory using URB597 in a rat model of endotoxemia showed comparable results with the exception of a therapeutic benefit seen in elevating mucosal villi and circular muscle FCD [32].

The most common MAGL inhibitor used experimentally* in vivo* is JZL184 [37, 38]. A recent study investigated MAGL knockout (K.O.) mice in a hepatic injury model [39]. The results showed wild-type mice given JZL184 and MAGL K.O. mice were protected from hepatic ischemia/reperfusion injury through increased CB_2_ signalling. JZL184 suppressed the oxidative stress and inflammation that resulted from ischemia/reperfusion by increasing endocannabinoid levels through MAGL inhibition. Our results with JZL184 showed similar findings with reduced inflammation indicated by less adherent leukocytes in both V1 and V3 venules. As seen with URB597, JZL184 administration showed no difference in muscular FCD to the LPS group, while mucosal villi FCD did not show significant improvement compared to LPS administration alone.

Our data indicate comparable results between HU308, URB597, and JZL184; however, we favour further testing with enzyme inhibitors over synthetic agonists for the following reasons. One advantage of using enzyme inhibitors is that potential side effects of synthetic agonist administration, for example, receptor desensitization or tachyphylaxis, can be minimized. Furthermore, hydrolysing enzymes work at local sites of endogenous cannabinoids production; therefore, a more precise activation of target receptors is possible due to localized increases in endocannabinoid levels. Disadvantages of using (unspecific) CB_2_ receptor agonists systemically are the potential activation of off-target receptors, for example, GPR55 [40]. With systemically elevated levels of endocannabinoids, alternative pathways like the eicosanoid pathway may be activated [41]. Arachidonic acid which is a metabolite of 2-AG is also a precursor molecule for prostaglandins and leukotrienes. In this study the use of JLZ184, a MAGL inhibitor, prevents 2-AG metabolism, thereby minimizing availability of arachidonic acid to be converted to leukotrienes and prostanoids. However, as an alternative, cyclooxygenases can oxygenate endocannabinoids which can then be hydrolyzed to prostaglandins. Endocannabinoid levels are relatively low even when enzyme inhibitors are employed, limiting the effect of the eicosanoid pathway in our study [41]. Another possible drawback of elevating endocannabinoid levels is their effects on off-target receptors like CB_1_ and GPR55. However, CB_1_-mediated effects, if present, would potentially increase leukocyte activation since inhibition of the CB_1_ pathway was shown by our group to reduce leukocyte adhesion to the endothelium in experimental endotoxemia [42]. GPR55 is highly expressed not only in the brain, but also in the gastrointestinal tract [43, 44]. Unfortunately, the role of GPR55 in intestinal inflammation has not yet been fully elucidated.

Although our acute endotoxin model was quite effective in producing an inflammatory response, this model has limited clinical relevance due to lack of pathogens in the body and lack of the multiphasic immune states evident during sepsis pathogenesis. Clinical sepsis in patients is usually triggered by an infecting pathogen; therefore, due to the lack of an active pathogen in our model, our results should be interpreted with some caution when translating to clinical relevance. More clinically relevant models of sepsis induce abdominal peritonitis through fecal translocation from the intestine [27, 45]. However, controversy still exists on the benefit of the CB_2_ pathway in these models [8]. Sepsis pathophysiology is quite complex in clinical settings, with time-dependent changes of the status of the immune system. Our study examined the immune state two hours after endotoxin administration thereby limiting our model to the initial proinflammatory phase of sepsis pathogenesis. Another limitation to our study is the possibility of a dose-dependent effect with our compounds. All our compound doses were based on previous dose response studies conducted in either rats or mice [29, 32, 46].

In conclusion, our results support the concept of targeting the CB_2_ pathway in sepsis to modulate the acute phase response of the immune system. We demonstrated the therapeutic benefit of the CB_2_ receptor agonist HU308 in reducing LPS-induced leukocyte recruitment within the intestinal microcirculation. Furthermore, an alternate mechanism of activating the CB_2_ pathway through the enzyme inhibitors URB597 and JZL184 produced similar results. In contrast, blocking CB_2_ receptors with AM630 caused additional FCD reduction in intestinal musculature. These results support the benefit of modulating the CB_2_ pathway in sepsis pathogenesis, warranting further investigation into this pathway in order to develop effective therapeutics.
